# Th1- and Th17-Related Cytokines in Venous and Arterial Blood of Sclerodermic Patients with and without Digital Ulcers

**DOI:** 10.1155/2019/7908793

**Published:** 2019-10-07

**Authors:** S. Nicola, M. Fornero, E. Fusaro, C. Peroni, M. Priora, G. Rolla, C. Bucca, L. Brussino

**Affiliations:** ^1^Università degli Studi di Torino, Dipartimento di Scienze Mediche—AO Ordine Mauriziano Umberto I, Torino, Italy; ^2^SC Reumatologia, AOU Città della Salute e della Scienza, Torino, Italy

## Abstract

The earliest clinical manifestation of SSc is usually Raynaud's phenomenon, a small-arteries vasospasm driven by vascular tone dysregulation and microcirculatory abnormalities, resulting in digital ulcers (DU) in up to 50% of patients. Many cytokines as well as growth factors have been shown to play a role in promoting vascular smooth muscle cell proliferation and fibroblast activation, leading to ischemic damage as well as skin fibrosis. We aim to investigate a possible difference in venous and arterial blood levels of many cytokines (Th1- and Th17-related), GM-CSF, and endothelin-1 (ET1) in patients with and without DU. In the same patients, the correlations between capillary damage, evaluated by nailfold videocapillaroscopy (NVC), extension of skin fibrosis, calculated by modified Rodnan skin score (mRSS), and cytokines, ET-1, and GM-CSF levels were also measured. Patients with DU showed venous levels of IL-1*β* (*p*=0.024), IL-6 (*p*=0.012), IL-22(*p*=0.006), and TGF-*β* (*p*=0.046) significantly higher compared to arterial levels and arterial levels of GM-CSF and TNF-alpha significantly higher compared to venous levels (*p* < 0.001). NVC abnormalities were correlated with arterial TNFa and venous IL22, IL23, and IL17 levels and negatively correlated with venous ET-1 levels, whereas mRSS showed a negative correlation with IL-21(*ρ* = −0.427, *p*=0.050). The increased Th17-cytokine levels in venous compared to arterial blood of patients with DU suggest local cytokine production on ulcer site. The higher TNFa and GM-CSF levels in arterial blood of DU patients support the attempt to mitigate the hypoxic damage, and the correlation between Th17-cytokines, mRSS, NVC, and ET1 agrees with the potent profibrotic stimulus at the onset of the disease, which decreases as the SSc progresses.

## 1. Introduction

Systemic sclerosis (SSc) is an autoimmune chronic connective tissue disease, affecting primarily the skin, characterized by a fibrotic involvement of many organs, such as the vascular and gastrointestinal system, lungs, and heart. Based on cutaneous involvement, SSc can be differentiated into two phenotypes, the diffuse SSc (dcSSc) and the limited one (lcSSc). The two phenotypes are also distinguishable on the basis of visceral involvement and autoantibodies pattern.

The earliest clinical manifestation of SSc is usually Raynaud's phenomenon (RP), a small-arteries vasospasm driven by vascular tone dysregulation and microcirculatory abnormalities, resulting in digital ulcers (DU) in up to 50% of patients [1–4].

The vascular damage is usually rapidly progressive, characterized by a recurrent activation and apoptosis of endothelial cells, with consequent intimal thickening, lumen stenosis, and vessel obliteration, leading to tissue hypoxia and to repeated ischemia-reperfusion cycles which induce progressive skin fibrosis.

It is well known that, as a response to vascular damage, endothelial cells release proinflammatory cytokines such as IL-6, IL-1*β*, and TNF-*α* and growth factors (GFs), among which are the vascular endothelial growth factor (VEGF) [5] and transforming growth factor *β* (TGF-*β*). These inflammatory mediators, in association with the profibrotic stimulus of the endothelin-1 (ET-1), promote leucocyte adhesion to the endothelium, vascular smooth muscle cell proliferation, and fibroblast activation. This cytokine milieu is observed particularly at sites of digital ulcers [6–8]. In fact, other cytokines related to Th-2, Th-17, and Th-22 have been shown to play a role in the development of capillary damage and fibrotic skin in patients with scleroderma [9].

Until now, no studies focused on sclerodermic digital ulcers have analyzed the different inflammatory patterns mirrored by cytokines measured in arterial and venous patients' serum.

The aim of our study was to evaluate proinflammatory TNF-*α*, Th-1- (IL-2) and Th-17- (IL1-*β*, IL-6, IL-9, IL-17, IL-21, IL-22, and IL-23) related cytokines, TGF-*β*, GM-CSF, and endothelin-1 (ET-1) in arterial and venous blood of patients affected by systemic sclerosis with and without DU and to analyze the correlations between cytokine levels and clinical scores of skin fibrosis and vascular involvement.

## 2. Methods

### 2.1. Patients

All consecutive outpatients attending the Clinic of Rheumatic Disease between May 2014 and March 2015, who received the diagnosis of SSc (according to the ACR criteria), were enrolled in the study; the exclusion criteria were smoking, asthma, COPD [10], history of cancer [11] or other autoimmune disease, current corticosteroid or immunosuppressive therapy, and current or recent (last 8 weeks) systemic or respiratory infection. Arterial and venous blood sampling, nailfold video capillaroscopy, and modified Rodnan skin score [12, 13] were obtained in all the patients. In patients presenting digital ulcers, both arterial and venous blood samples were taken from the same side as the ulcers. Our Institutional Review Board for human studies approved the study protocol (no. 0039653), and the study respected the Helsinki Declaration.

Venous blood samples were obtained in twenty healthy nonsmoking subjects, who served as controls.

### 2.2. Cytokine Assays

Arterial and venous blood cytokines were analyzed by multiplex immunoassay (Bio-Rad Laboratories Inc., Hercules, CA, USA), with Bioplex 100 xMAP technology (Luminex Corp, Austin, TX, USA), and Bioplex Manager 4.1 software (Bio-Rad Laboratories, Segrate, Italy) was used for the data analysis. Every 96-well plate included an 8-point standard curve, and the same assay plate was used for patient and control samples.  Table 1 reports the percentage of samples above the detection threshold. Low cytokine concentrations measured not on the linear part of the standard curves were considered below the limit of detection [14, 15]. Further analysis was performed on data set which included parameters (cytokines and growth factors) measured at concentrations higher than the detection limits in over 50% of samples.

### 2.3. Nailfold Videocapillaroscopy (NVC)

Nailfold videocapillaroscopic examination was performed with a epiluminescence video-bio-microscope for immersion microscopy and polarized light microscopy (Videocap, DS Medica, Milan, Italy). Each test was evaluated by two different rheumatologists (CP and EF) using a qualitative score based on the morphology of nailfold capillaries. The patterns identified within the “scleroderma pattern” included (1) “early” NVC pattern: few enlarged/giant capillaries, few capillary hemorrhages, mostly well-preserved capillary distribution, and no evident loss of capillaries; (2) “active” NVC pattern: frequent giant capillaries, frequent capillary hemorrhages, moderate loss of capillaries, mild disorganization of the capillary architecture, and absent or mild ramified capillaries; (3) “late” NVC pattern: irregular enlargement of the capillaries, few or absent giant capillaries and hemorrhages, severe loss of capillaries with extensive avascular areas, disorganization of the normal capillary array, and ramified/bushy capillaries [16].

### 2.4. Modified Rodnan Skin Score (mRSS)

Rodnan skin score, a semiquantitative score based on the fibrotic involvement of the skin, according to Khanna et al. [13] was obtained from all of the patients.

The score ranges from 0 to 51, being analyzed in 17 different areas of the body surface, some of which considered as double. Each area received a score ranging from 0 to 3, on the basis of the following criteria: mRSS = 0 was “normal skin” with fine wrinkles but no skin thickness; mRSS = 1 indicated “mild” skin thickness with possible folding between 2 fingers; mRSS = 2 corresponded to “moderate” skin thickness with difficulty in making skin folds and no wrinkles. mRSS = 3 indicated “severe” skin thickness with inability to make skin folds between 2 examining fingers.

### 2.5. Statistical Analysis

The statistical analysis was performed using a commercially available statistical package (STATA 10s), and only *p* values <0.05 were considered statistically significant.

Based on normality distribution tests (Kolmogorov–Smirnov, Shapiro–Wilk, and D'Agostino's K-squared), most cytokine concentrations were not normally distributed in both patients and healthy controls.

A comparison between arterial and venous blood was performed using the nonparametric test for independent or paired samples, based on the cohort of patients (healthy controls or patients' group).

The correlations between arterial or venous cytokines and clinical (mRSS) and instrumental (NVC) parameters were analyzed by regression analysis. Spearman's rank correlation coefficient was calculated according to the nonnormal distribution of data.

## 3. Results

Twenty-nine patients affected by SSc (28 females and 1 male) were enrolled in the study, as well as 20 healthy controls (12 females and 8 males).

The mean age was 64.5 years (range 28–80) in patients and 59 years (range 47–65) in healthy subjects (n.s.). Patients were then divided into two groups, based on the presence of digital ulcers (20 patients) or not (9 patients). All cytokines were detectable both in arterial and venous blood samples in patients and in venous blood samples of controls.

### 3.1. Comparison between Venous Blood Concentration of Cytokines in Patients and Controls

Serum levels of all the cytokines and ET-1 were significantly higher in the patients compared to healthy controls, except for IL-9 and IL-13 (Table 1).

### 3.2. Comparison between Arterial and Venous Blood Cytokine Concentration

A significantly higher concentration of IL-1*β* (*p*=0.024), IL-6 (*p*=0.012), IL-22 (*p*=0.006), and TGF-*β* (*p*=0.046) was observed in venous compared to arterial blood samples only in patients with DU, who also showed a significantly higher concentration of GM-CSF and TNF-alpha in arterial compared to venous blood samples (*p* < 0.001) (Table 2).

No significant differences were observed between arterial and venous cytokine concentrations in patients without digital ulcers (Table 2).

### 3.3. Correlations between Cytokines and Clinical Parameters of Capillary (NVC) and Skin (mRSS) Involvement

Venous IL-22 (*ρ* = 0.460, *p*=0.041), IL-23 (*ρ* = 0.411, *p*=0.042), and IL-17 (*ρ* = 0.465, *p*=0.039) concentrations were positively correlated and ET-1 (*ρ* = −0.437, *p*=0.044) inversely correlated with NVC pattern in patients with DU, who also showed a significant correlation between NVC and arterial TNF-*α* concentration (*ρ* = 0.460, *p*=0.045). A negative correlation between mRSS and venous IL-21 (*ρ* = −0.427, *p*=0.050) was observed (Table 3).

No correlations were observed between serum cytokines (arterial nor venous) and skin involvement in patients without digital ulcers.

## 4. Discussion

Th1 and Th17 cytokines, as well ET-1, serum levels were significantly higher in patients compared to controls, indicating a systemic inflammatory status in patients who were not receiving any immunosuppressive drugs.

Looking at the patients with DU, it is interesting that they showed higher concentration of inflammatory (IL-6), TGF-*β*, and Th17 (IL-1*β*, IL-22) related cytokines measured in venous compared to arterial blood, while no differences could be appreciated in patients without DU.

This observation suggests a local production of Th1- and Th17-related cytokines, which drive a proinflammatory and profibrotic tissue response.

Actually, Th17-related cytokines, particularly IL-17A, are thought to be involved in the pathogenesis of skin lesions in SSc by inducing adhesion molecules production and promoting collagen synthesis and proliferation [17]. Local skin production of Th1- and Th17-related cytokines has been found by Brembilla et al. [18], who also showed an increase of Th-22 cells in peripheral blood of patients affected by SSc, together with an increase of IL-22 mRNA in skin biopsies, suggesting a potential role of Th-17 and Th-22 in the pathogenesis of tissue fibrosis [18–20]. In our patients, we observed a significant correlation between Th17-related cytokines and capillary damage evaluated by NVC. In the same patients, we showed an inverse correlation between ET-1 levels and NVC scleroderma pattern. This observation may be explained by the well-known profibrotic stimulus played by ET-1 at the onset of disease and its reduction as the SSc progresses.

It has indeed been shown by Chora et al. that high serum ET-1 levels were strongly related to lung and vessel fibrosis only in patients with active SSc and not in the very early or late phases of disease [21].

Our results also found a negative correlation between venous IL-21 and mRSS in patients presenting DU. Zhou and colleagues demonstrated that mRNA of IL-21 was higher in the early phase of SSc, postulating IL-21 could be a biomarker able to identify lesions severity in early SSc [22].

The negative correlation we found between IL-21 and mRSS perfectly fits with the abovementioned finding, due to the elevated inflammation on the ulcer's site at the onset of disease and due to the reduction in cytokine recruitment when skin fibrosis plays a leading role and mRSS is higher.

The higher TNF-*α* and GM-CSF concentrations we found in the arterial blood samples of patients with DU could mitigate the hypoxic tissue damage, according to the physiological function of these cytokines. It has been demonstrated that the synthesis of TNF-*α* is increased immediately after ischemic injury, where it plays a role in regulating cells survival or apoptosis and in driving cellular inflammatory responses [5, 23]. Likewise, GM-CSF is a cytokine with pleiotropic functions, ranging from the regulation of proliferation, differentiation, and survival of hematopoietic cells to the mobilization and recruitment of hematopoietic and endothelial stem cells from the bone marrow. It also acts as a differentiation promoter of endothelial cells and fibroblasts and as a stimulatory factor for keratinocyte proliferation. To date, the efficacy of GM-CSF intradermal administration in the treatment of chronic venous leg ulcers is well known [24].

In conclusion, our study provides new information, which might suggest further possible targeted therapeutic approaches for digital ulcers of patients with scleroderma, who are presently lacking in effective approved therapies, except for the antagonists of ET-1.

## Figures and Tables

**Table 1:** Cytokine concentrations (pg/ml) in venous blood of patients affected by SSc and in healthy controls.

	Patients median [CI 95%]	Healthy controls median [CI 95%]	*p*
TNF-*α*	2.07 [0.21–8.72]	1.22 [0.87–1.75]	0.008
IL-2	14.57 [3.52–18.42]	6.33 [1.62–22.73]	<0.001
IL-5	0.30 [0.05–10.76]	0.08 [0.00–3.38]	<0.001
IL-9	0.43 [0.11–11.89]	0.90 [0.10–34.25]	n.s.
IL-13	2.92 [0.42–76.85]	2.00 [0.11–9.50]	n.s.
GM-CSF	140.96 [138.65–141.42]	22.28 [20.72–22.56]	<0.001
IL-23	8.84 [0.60–14.79]	1.62 [1.58–1.72]	<0.001
IL-1b	0.18 [0.03–1.22]	0.32 [0.20–0.97]	<0.001
IL-6	3.82 [0.36–30.02]	1.23 [0.12–4.68]	0.011
IL-17	1.08 [0.41–1.65]	0.54 [0.04–0.85]	0.041
IL-21	23.38 [3.73–38.33]	3.60 [3.51–3.87]	<0.001
IL-22	3.75 [0.68–6.48]	1.95 [0.59–6.37]	<0.001
ET-1	15.67 [3.41–48.68]	5.42 [3.32–6.63]	<0.001
TGF-*β*	6.62 [5.03–26.24]	4.45 [3.82–14.64]	<0.001

**Table 2:** Comparison between venous and arterial blood cytokine concentrations in patients with and without digital ulcers.

Cytokines	Patients with digital ulcers			Patients without digital ulcers		
	Venous concentration median (pg/ml) [CI 95%]	Arterial concentration median (pg/ml) [CI 95%]	*p*	Venous concentration median (pg/ml) [CI 95%]	Arterial concentration median (pg/ml) [CI 95%]	*p*
TNF-*α*	8.51 [0.21–8.72]	28.81 [0.06–28.87]	<0.001	3.78 [2.06–7.08]	1.64 [ 0.37–4.03]	n.s.
IL-2	14.47 [9.95–15.03]	15.30 [11.77–15.86]	n.s.	15.24 [12.81–16.70]	16.02 [7.43–17.20]	n.s.
IL-5	0.33 [0.09–1.63]	0.19 [0.05–1.19]	n.s.	0.30 [0.13–4.20]	0.22 [0.02–4.53]	n.s.
IL-9	0.65 [0.11–3.31]	0.36 [0.13–2.47]	n.s.	0.42 [0.11–1.32]	0.37 [0.17–0.62]	n.s.
GM-CSF	140.88 [140.26–140.99]	141.18 [140.81–141.28]	<0.001	140.99 [139.97–141.32]	141.27 [140.84–141.48]	n.s.
IL-23	10.73 [7.14–11.62]	10.73 [10.58–11.68]	n.s.	8.81 [4.80–10.51]	9.60 [8.49–10.74]	n.s.
IL-1*β*	0.20 [0.17–0.64]	0.14 [0.11–0.16]	0.024	0.18 [0.03–0.67]	0.18 [0.11–0.21]	n.s.
IL-6	3.71 [2.52–11.03]	1.64 [1.22–3.98]	0.012	2.09 [1.23–4.32]	1.95 [0.63–3.01]	n.s.
IL-17	1.08 [0.78–1.18]	1.12 [0.94–1.33]	n.s.	1.17 [0.80–1.25]	0.93 [0.48–1.34]	n.s.
IL-21	23.38 [17.81–27.00]	23.38 [21.42–27.01]	n.s.	23.38 [14.55–27.49]	25.36 [17.04–30.01]	n.s.
IL-22	4.64 [4.13–5.30]	3.62 [3.33–4.60]	0.006	4.52 [3.42–5.23]	5.26 [4.43–5.70]	n.s.
TGF-*β*	7.10 [6.31–13.10]	7.02 [6.44–7.06]	0.046	6.24 [3.96–11.27]	6.62 [6.17–7.13]	n.s.
ET-1	14.39 [13.51–16.38]	15.88 [11.38–17.40]	n.s.	15.88 [9.36–28.08]	17.37 [10.76–24.55]	n.s.

**Table 3:** Correlations between capillary (NVC) and skin (mRSS) involvement and blood cytokine concentration in patients with digital ulcers.

Cytokines	Nailfold videocapillaroscopy	
Venous blood		
IL-17	*ρ* = 0.465	*p*=0.039
IL-22	*ρ* = 0.460	*p*=0.041
IL-23	*ρ* = 0.411	*p*=0.042
ET-1	*ρ* = −0.437	*p*=0.044

Arterial blood		
TNF-*α*	*ρ* = 0.460	*p*=0.045

	mRSS	
Venous blood		
IL-21	*ρ* = −0.427	*p*=0.050

## Data Availability

All data are available if requested.
